# Rarity of molt evidence in early pennaraptoran dinosaurs suggests annual molt evolved later among Neornithes

**DOI:** 10.1038/s42003-023-05048-x

**Published:** 2023-07-03

**Authors:** Yosef Kiat, Jingmai Kathleen O’Connor

**Affiliations:** Negaunee Integrative Research Center, Field Museum of Natural History, 1400 S DuSable Lake Shore Drive, Chicago, IL 60605 USA

**Keywords:** Palaeontology, Palaeoecology

## Abstract

Feathers are a primitive trait among pennaraptoran dinosaurs, which today are represented by crown birds (Neornithes), the only clade of dinosaurs to survive the end Cretaceous mass extinction. Feathers are central to many important functions and therefore, maintaining plumage function is of great importance for survival. Thus, molt – by which new feathers are formed to replace old ones, is an essential process. Our limited knowledge regarding molt in early pennaraptoran evolution is based largely on a single *Microraptor* specimen. A survey of 92 feathered non-avian dinosaur and stem bird fossils did not find additional molting evidence. Due to its longer duration, in ornithological collections evidence of molt is found more frequently in extant bird species with sequential molts compared to those with more rapid simultaneous molts. The low frequency of molt occurrence among fossil specimens resembles collections of bird species with simultaneous molts. The dearth of molt evidence in the forelimbs of pennaraptoran specimens may have interesting implications regarding molt strategy during early avian evolution, and suggests that the yearly molting cycle may have evolved later, among crown birds.

## Abstract (web summary)

In this perspective, Kiat and O’Connor review non-avian dinosaur and stem bird fossils data and propose that annual molt evolved among Neornithes at a later point than early pennaraptoran dinosaurs.

## Introduction

**Fig. 1:**
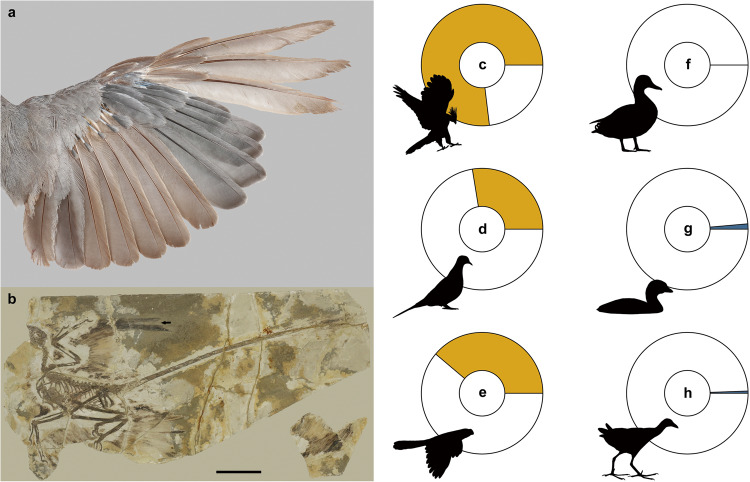
Primary feathers molt in birds and *Microraptor*. **a** An active primary molt includes a temporary molt-related gap in the wing flight surface (White-throated Robin, *Irania gutturalis*). **b** Primary sequential molt in *Microraptor* (IVPP V13352). The arrow indicates the location of the molt-related gap within the primary feathers^4^, scale bar equals 10 cm. **c**–**e** The proportion of actively molt specimens in three species performing sequential primary molt: **c** Hoatzin (*Opisthocomus hoazin*; 76.9%, *n* = 52 specimens), **d** Mourning Dove (*Zenaida macroura*; 27.6%, *n* = 127), and **e** Smooth-billed Ani (*Crotophaga ani*; 38.8%, *n* = 103), and **f**–**h** three species performing simultaneous primary molt: **f** Mallard (*Anas platyrhynchos*; 0.0%, *n* = 81), **g** Pied-billed Grebe (*Podilymbus podiceps*; 1.4%, *n* = 72), and **h** Purple Gallinule (*Porphyrio martinica*; 0.6%, *n* = 175). These data demonstrate the relationship between the molt duration and the chance of finding an individual in an active molt. Species in which the molt duration is longer (for example, sequential molt) will show a higher proportion of specimens with an active molt compared to species in which the molt duration is shorter (for example, simultaneous molt).

Vaned feathers, which evolved among pennaraptoran theropod dinosaurs and were inherited by crown birds (Neornithes), are involved in many important functions. These include locomotion (flight, aerial maneuverability, swimming and buoyancy), thermoregulation, visual communication, camouflage, protection from solar radiation and parasites, and waterproofing^1,2^. One interesting and important characteristic of feathers is that, unlike other keratin-based structures, full-grown feathers become incapable of growth or renewal. Therefore, the only way to renew a damaged feather is to replace it, which causes a temporary gap in the plumage^1,3^. The process by which new feathers are formed to replace old ones is called molt (Fig. 1a), and like feathers themselves, it evolved among dinosaurs long before the appearance of crown birds (Fig. 1b)^4^.

In addition to partial molts that allow for breeding or seasonal differences in plumage in some species (e.g., in plumage coloration)^5^, birds experience a single complete molt annually in which the entire plumage is renewed^1^. Most or all bird species seem to adhere to this roughly annual molt cycle, even in the tropics where molt may respond to annual rainfall regimes. There is little evidence for consistent molt cycles of more or less than a year in species of wild birds, although individuals may experience some variability between years. This fixed trait is most likely a result of the great importance of molt and the potential cost involved in avoiding plumage renewal. Due to the lack of variation among birds, nothing is known about the evolutionary development of this trait or when the annual molting cycle first evolved.

## The rarity of molt evidence in early pennaraptoran dinosaurs

In addition to the lack of variation among neornithines, the rarity of molt documentation in non-avian dinosaurs apparently also does not contribute to our understanding of the appearance of the annual molt cycle. To date, only one record of active molting among non-neornithine pennaraptorans has been identified^4,6^; all other evidence of growing feathers refers to young individuals exhibiting pre-juvenal molt^7–9^ which is not relevant here.

Here, we propose that the rarity of molt evidence in non-avian dinosaurs and stem birds may hint at the evolutionary history of this trait. Specifically, we hypothesize that given complete randomness in our access to specimens, a correlation is expected between species-specific molt duration and the number of individuals that will be recorded in active molt out of a random pool of specimens. Taking a broader view, we predict that in a random sample of individuals belonging to species performing a sequential molt, a gradual replacement of the flight feathers carried out over an extended duration, we will find more individuals in active molt than in a sample of individuals belonging to a species performing simultaneous molt, which occurs over a shorter duration.

In order to test this prediction, we examined specimens (skins) of six bird species stored in the Field Museum of Natural History (Chicago, USA). Three species have sequential molts (long molt duration): (1) Hoatzin (*Opisthocomus hoazin*; *n* = 52 specimens), (2) Mourning Dove (*Zenaida macroura*; *n* = 127), and (3) Smooth-billed Ani (*Crotophaga ani*; *n* = 103), and three species have simultaneous molts (short molt duration): (4) Mallard (*Anas platyrhynchos*; *n* = 81), (5) Pied-billed Grebe (*Podilymbus podiceps*; *n* = 72), and (6) Purple Gallinule (*Porphyrio martinica*; *n* = 175). For each specimen, we visually tested whether an active primary molt is present or absent. The results of this test corresponded to the prediction: the three species characterized by long molt duration (sequential molt) showed a significantly higher proportion of individuals in active molt compared to the three species characterized by short molt duration (simultaneous molt; χ^2^ = 155.26, df = 1, *P* < 0.001; Pearson’s χ^2^ test; Fig. 1c–h).

In order to examine this proportion among non-avian dinosaurs and stem birds, we tested a large sample of pennaraptoran fossils (*n* = 92 specimens; Supplementary Data 1) and determined how many of these we could define as almost certainly not having died during active primary molt. This sample constitutes all specimens preserving feathers available at the Institute of Vertebrate Paleontology and Paleoanthropology (IVPP), as well as those published in the scientific literature. This sample is also considered random as there is no information to suggest seasonality in the deaths of these individuals and, while collections may be biased towards feathered specimens, there is no bias in favor of or against individuals in molt, a subtle feature undetectable to the untrained eye. The results suggested that for 65 of these specimens we could not determine whether or not active molting was present at time of death; 26 fossil specimens almost certainly do not preserve an active molt; and only *Microraptor* IVPP V13352 preserves an active primary molt, as documented previously^4^. In this *Microraptor* specimen, primary feather molt was identified by the distinct gap visible in the right wing which is created by three gradually growing primary feathers (Fig. 1b). This proportion of molt among fossil specimens does not differ from the three species performing simultaneous and short duration molt (Mallard: χ^2^ = 0.34, *P* = 0.56, Pied-billed Grebe: χ^2^ < 0.01, *P* = 1.00, and Purple Gallinule: χ^2^ = 0.24, *P* = 0.63), but significantly differs from those three performing sequential and long duration molt (Hoatzin: χ^2^ = 35.29, *P* < 0.001, Mourning Dove: χ^2^ = 5.81, *P* = 0.02, and Smooth-billed Ani: χ^2^ = 10.66, *P* < 0.01).

Molt duration, which affects the proportion of individuals with an active molt among a random sample of individuals, is mainly derived by the molt rate^10^. Molt rate is a continuous variable determined by the rate of feather growth; even among the species defined here as having sequential molt, variation is mainly in the number of primaries replaced at the same time^1^. This variation is mainly the result of species-specific differences in dependence on flight ability^11^ and the duration of time available for molt^1^. Body size also has an effect with larger species having longer molt duration because of the larger size of their feathers^10^.

We offer two possible explanations for these results. (1) Simultaneous molt was more common among pennaraptoran dinosaurs (including stem birds) than previously thought. Aerodynamic abilities inferred present in some non-avian dinosaur lineages and inherited by birds^12–16^ led to the development of two remex molt strategies observed in neornithines: sequential and simultaneous. Previous analysis has suggested that the sequential feather molt evolved in Paraves at least 50 myr prior the appearance of the crown birds^4^ and that simultaneous molt evolved at the earliest about 50 mya (among neornithines). Simultaneous molt most likely arose among large bodied taxa or those living in protected habitats, such as waterfowl, probably as an adaptation for reducing the duration of remex molt^4^. Thus, in contrast to the non-sequential (and non-simultaneous) molt, which has primarily evolved in some flightless species (e.g., Common Ostrich *Struthio camelus*, Flightless Cormorant *Nannopterum harrisi*, and Kākāpō *Strigops habroptila*) and thus may have been common among early non-volant pennaraptorans, the simultaneous molt would not be expected to be very common among extinct volant taxa, and thus, this hypothesis is not very likely. Alternatively (2), non-avian pennaraptoran dinosaurs had a sequential primary molt but their molting cycle was longer than one year, i.e., the frequency of molt was lower than among extant birds. The result is that each individual spends less of its life time molting, and therefore, the proportion of individuals experiencing active molt from among a random sample of individuals will be low compared to species with an annual molting cycle (all extant neornithines). The second hypothesis may also be supported by the fact that feathers that appear highly abraded, as would be expected if molt was less frequent (i.e., a molt cycle longer than a year), are common in Cretaceous pennaraptoran fossils (e.g., *Caudipteryx* NGMC-97-4, *Microraptor* BMNHC-PH881, *Protopteryx* STM7-143, *Sapeornis* DNHM-D3078). However, we will qualify this by saying that our observations regarding feather abrasion is based on extant birds and the rate of feather abrasion in early taxa may have been different. It is also possible that what appears to be abrasion may in fact be the result of the fossilization process and not indicate in vivo plumage abrasion. Unfortunately, studies quantifying feather abrasion in birds are rare; thus, future research will be required to define comparable methods for measuring feather abrasion that can be used to compare modern birds and fossil specimens. Moreover, the basic premise presented here may be incorrect—it may be that our sample is not random and therefore does not correctly reflect the proportion of actively molting individuals in the entire population. Such a bias may arise in modern bird skin specimens as a result of the collector’s preference for non-molting birds which may be more perfect for exhibition and simpler to prepare. Similarly among fossils, if for example, individuals who avoid molting for some reason (i.e., to reduce energetic costs) tended to die and fossilize at a higher rate than their proportion in the real population, or if these specimens, which do not show active molt, died seasonally at a time that did not coincide with molt period, the result of both examples would be that molt would be underrepresented in our sample. However, at this time there is no evidence that indicates either sample is in fact non-random.

Our hypothesis suggests that the evolution of the flight feather annual molt evolved with the development of powered flight, among crown birds or a more derived subset of pennaraptorans (i.e., Ornithurae), and as a response to the high dependence of this group on the flight feathers and the aerodynamic ability they impart. This strategy probably improves the ability to maintain high performance of the feathered flight surface in dinosaurs with true powered flight throughout the yearly cycle. The data gleaned from this statistical comparison of extant and extinct collections provides an intriguing hypothesis regarding molting in early pennaraptorans with limited flight capabilities. Further integration between ornithology neontological and paleontological data^16^ and additional fossils will shed new light on the subject discussed here and improve our understanding of basic avian life-history traits evolutionary processes.

## Supplementary information

Description of Additional Supplementary Files

Supplementary Data 1

## Data Availability

All data supporting the study findings were provided as Supplementary material.
