# Supplementary material for: Rarity of molt evidence in early pennaraptoran dinosaurs suggests annual molt evolved later among Neornithes
Source: Commun Biol. 2023 Jul 3;6:687. doi: 10.1038/s42003-023-05048-x (PMC10317961; doi:10.1038/s42003-023-05048-x)
Supplement: Supplementary file 1 — Description of Additional Supplementary Files [file 42003_2023_5048_MOESM1_ESM.pdf]

# Description of Additional Supplementary Files

**File name:** Supplementary Data 1
**Description:** The list of specimens tested in the study and the presence (1) or absence (0) of the molt.
